# Colorectal Cancer Screening Based on Predicted Risk: A Randomized Controlled Trial

**DOI:** 10.14309/ajg.0000000000003311

**Published:** 2025-01-07

**Authors:** Ekaterina Plys, Jean-Luc Bulliard, Aziz Chaouch, Marie-Anne Durand, Luuk A. van Duuren, Karen Braendle, Reto Auer, Florian Froehlich, Iris Lansdorp-Vogelaar, Douglas A. Corley, Kevin Selby

**Affiliations:** 1 Unisanté, University Center for Primary Care and Public Health, University of Lausanne, Lausanne, Switzerland;; 2 The Dartmouth Institute for Health Policy & Clinical Practice, Dartmouth College, Lebanon, New Hampshire, USA;; 3 Department of Public Health, Erasmus University Medical Centre, Rotterdam, the Netherlands;; 4 Institute of Primary Health Care (BIHAM), University of Bern, Bern, Switzerland;; 5 Department of Gastroenterology, University Hospital of Basel, Basel, Switzerland, and Jura district Hospital, Porrentruy, Switzerland;; 6 Division of Research, Kaiser Permanente Northern California, Oakland, California, USA.

**Keywords:** colorectal cancer screening, personalized screening, risk communication, organized screening, White, Switzerland

## Abstract

**INTRODUCTION:**

Colorectal cancer (CRC) screening relies primarily on colonoscopy and fecal immunochemical testing (FIT). Aligning utilization of these options with individual CRC risk may optimize benefit with lower risks, individual burden, and societal costs. We studied the effect of communicating personalized CRC risk and corresponding screening recommendations on risk-appropriate screening uptake in an organized screening setting.

**METHODS:**

Randomized controlled trial among residents aged 50–69 years not yet invited for screening in Vaud, Switzerland. The intervention was a mailed brochure communicating individual 15-year CRC risk and screening recommendation. The control group received a usual brochure comparing FIT and colonoscopy. The primary outcome was self-reported risk-appropriate screening (FIT if <3% risk, FIT or colonoscopy if ≥3% and <6%, and colonoscopy if ≥6%) at 6 months. A secondary outcome was overall screening uptake.

**RESULTS:**

Of 5,396 invitations, 1,059 people responded (19%) of whom 258 were randomized to intervention and 257 to control materials (average 15-year risk 1.4% [SD = 0.5], age 52.2 years [SD = 2.2], 51% women). Risk-appropriate screening completion was 37% in the intervention group and 23% in the control group (absolute difference 14%, 95% confidence interval 6%–22%). Overall screening uptake was 50% in the intervention group and 49% in the control group (absolute difference 1%, 95% confidence interval −7% to 10%).

**DISCUSSION:**

In a population not known to be at elevated CRC risk, brochures providing personalized CRC risk and screening recommendations improved risk-appropriate screening without impacting overall screening uptake. This approach could be helpful for aligning screening methods, risks, and benefits with cancer risk and resource allocation.

## INTRODUCTION

Colorectal cancer (CRC) is the third most common cancer in men and second most common in women, causing approximately 930,000 deaths worldwide every year (1). The long preclinical development of the disease allows for screening to reduce CRC mortality (2). In Switzerland, 48% of the population aged older than 50 years was up-to-date with screening in 2017, and 43% had had a screening or diagnostic colonoscopy within the past 10 years (3). However, there is no direct evidence for the superiority of colonoscopy compared with fecal immunochemical tests (FITs) in an organized screening setting to detect CRC (4) and reduce CRC mortality (2), especially among individuals at low or average risk.

Personalized CRC screening could decrease overuse of colonoscopy by reserving this method for individuals at high risk and orienting others to FIT. Personalized screening includes estimating individual risk and providing corresponding screening recommendations. Personalized screening to decide who should or should not be offered any CRC screening has not been widely accepted; risk-based recommendations between colonoscopy and FIT are more in-line with current trends. A recent review and guideline statement concluded that this approach could diminish harms related to screening, the burden of colonoscopy preparation, and free-up colonoscopy resources in areas with widespread use of screening colonoscopy, but that evidence is lacking regarding its implementation (5,6).

The impact of personalized screening recommendations on screening behaviors is not clearly understood. Most studies have tested its influence on overall screening uptake (7–11), and only 2 studies focused on risk-appropriate screening, meaning use of colonoscopy by those at high risk and noninvasive tests by those at low risk (12,13). Results of these studies suggest that communicating risk without personalized screening recommendations is insufficient to influence individual decision-making (13). The primary aim of this trial was to study whether communicating individual CRC risk and screening recommendations with written materials affects risk-appropriate screening uptake at 6 months. We hypothesized that participants at low risk would be more likely to choose FIT and participants at high risk colonoscopy.

## METHODS

This study followed the CONSORT Guidelines for reporting outcomes in trial reports (14). The trial was registered before inclusion of the first participant, and the study protocol was later published (15).

### Trial design

This was a monocentric, 2-arm randomized controlled superiority trial with participants randomized 1:1 in the intervention and control arms. It also investigated the feasibility of a clinical trial in the context of an organized screening program. The trial was nested in the CRC screening program of the canton of Vaud, Switzerland, that offers to eligible individuals a choice between FIT and colonoscopy (16).

### Participants

**Figure 1.**
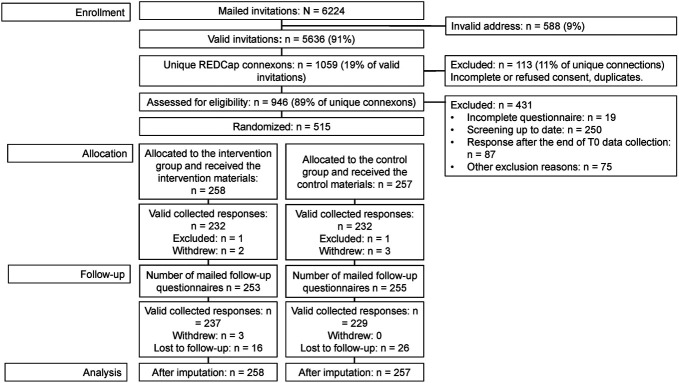
Flowchart of participants.

Recruitment took place between June and September 2022. To not interfere with existing screening routines, we contacted people aged 50–69 years who had not yet been invited to the Vaud screening program. Individuals were excluded if they had symptoms suggestive of CRC; personal history of CRC, advanced adenoma, or inflammatory bowel disease; genetic syndromes representing high risk for CRC; if they were up-to-date with screening (colonoscopy within 9.5 years or FIT within 1.5 years); or expected to leave Switzerland within 6 months. See Figure 1 and Supplementary Figure 1 (Supplementary Digital Content 1, http://links.lww.com/AJG/D529) for details.

### Intervention and comparator

The intervention consisted of mailing participants a brochure containing their 15-year CRC risk and corresponding screening recommendations. The individuals at low risk were recommended FIT, those at high risk were recommended colonoscopy, and those at moderate risk did not get a specific recommendation.

The intervention was based on the health belief model (17). It was expected that, after the intervention, people at high risk would feel more susceptible and would complete colonoscopy, whereas people at low risk would feel less susceptible and prefer FIT. Screening recommendations were expected to increase self-efficacy. Brochures for those at low risk informed them that zero risk did not exist and strongly recommended a screening test. As there are no strong recommendations for individuals at moderate risk, the brochure presented FIT and colonoscopy as equal options.

The control brochure was the standard brochure used by the Vaud screening program, which presented the advantages and disadvantages of both FIT and colonoscopy. It did not include risk-based screening recommendations (15). For more information about intervention and control materials (see Supplementary Table 1, Supplementary Digital Content 2, http://links.lww.com/AJG/D530).

Individual CRC risk scores were calculated using the QCancer-colorectal 15-year risk calculator (https://qcancer.org/15yr/colorectal/) (18). This open-source tool, developed and validated in the United Kingdom, assigns individuals aged 40–69 years old to the correct risk group 66%–70% of the time, which ranks QCancer among tools with the highest discriminative power (18–21). We slightly modified the calculator as follows. As people having advanced adenoma and inflammatory bowel disease were ineligible, it was stated “no ulcerative colitis” and “no colonic polyps” for all participants. In addition, as postcode and ethnicity have not been validated for use in Switzerland, we indicated “white or not stated” and left the postcode field blank. Our final version of the QCancer algorithm included the following variables: age, sex, tobacco and alcohol status, body mass index, type 2 diabetes, family history of CRC, and prior diagnoses of several cancers (see (15) for details). Based on the obtained risk score, individuals were divided in 3 risk levels: low risk (<3%), moderate risk (3%–5%), and high risk (≥6%), based in part on the 3% threshold of the BMJ Rapid Recommendation (22).

### Recruitment and data collection procedures

The study included 3 phases. At T0, 6,200 invitations with consent and the recruitment questionnaires were mailed, allowing us to verify eligibility and calculate CRC risk. At T1, the enrolled participants received intervention or control materials, questionnaire 2, and an information sheet to facilitate discussions with health professionals. At T2, 6 months after the intervention, screening behavior was measured using the follow-up questionnaire. Because mailings and return can take several weeks, responses were accepted up to 8 months after the intervention. The consent and all 3 questionnaires were available in paper and electronic form. All study documents were written in French. For more details, see our study protocol (15).

### Outcome measures

Our primary outcome was self-reported risk-appropriate screening uptake measured 6 months after the intervention. Screening was considered appropriate when participants at low risk completed FIT, those at high risk completed colonoscopy, and those at moderate risk completed colonoscopy or FIT. We considered a colonoscopy appointment as a completed test because waiting time for colonoscopy can take several months and missed screening appointments are rare (23).

### Secondary outcomes

Overall screening participation (at T2) was calculated as the proportion of individuals who completed any screening test.

Anxiety related to the printed materials (at T1) was assessed using 6 items adapted from the Spilberger's State-Trait Anxiety Inventory (24). Participants were asked to respond to these items after having read the brochure.

### Quality assurance, citizen engagement, and ethical considerations

Only validated questionnaires or questionnaires pretested in previous studies were used. After entering data in REDCap (25), 20% of responses collected using paper questionnaires were double checked. Six individuals of the target population (a citizen advisory group) were involved in development of the materials for participants, and in interpretation and dissemination of the results. The trial was monitored by an institutional monitoring team and received approval from the Ethics committee of the canton of Vaud on March 2, 2022 (project ID 2021–02431).

### Sample size

It was expected that 60% of the individuals eligible for screening would be at low risk, 10% at high risk, and 30% at moderate risk. Our intervention was expected to increase risk-appropriate screening in low-risk and high-risk participants, but not in those at moderate risk. To detect a difference of 10% in risk-appropriate screening between groups with power of 80% and an alpha of 5%, we needed 393 individuals in each group. After considering attrition of about 10%, the final sample size was estimated at 440 individuals in each group (880 in total). See our study protocol (15) for details.

During recruitment, we realized that nearly all eligible participants were at low risk. We decided to focalize on the available population and recalculated sample size. With 95% of participants at low risk, we would need 451 participants in total to have 80% power to detect a 15% change in risk-appropriate screening.

### Randomization

The REDCap automatic randomization module was programmed with the block factor varying between 4 and 8. Randomization was performed after entering the risk score by clicking on the button “randomize.” The trial team member who performed the randomization was not blinded. Participants were told that the study aimed at comparing 2 brochures on CRC screening and, therefore, were not aware of group assignment. The trial statistician who conducted the primary outcome analyses was blinded to participants' group assignments.

### Statistical methods

Intention-to-treat analyses were conducted. Randomization quality was tested using a 2-tailed χ^2^ or Fisher exact tests for categorical variables; Student *t* test or Mann-Whitney U tests were used for continuous variables. The primary outcome was analyzed using the 2-tailed χ^2^ test. Participants who refused screening and those who did not respond to the follow-up questionnaire were treated as not having performed a screening test in both groups. Prespecified subgroup analyses for the main outcome were performed by sex, nationality, education, occupation, French proficiency level, household size, family history of CRC, and polyps using a proportion test. The overall participation was tested using the 2-tailed χ^2^ test. Analyses of all secondary outcomes were conducted using a 2-tailed *t* test. Data analyses were performed using the Stata 18 (26) and R software packages (27).

### Important changes to methods after trial commencement

After trial commencement, several modifications to the protocol were made. Given the lower-than-expected response rate, 2,000 additional invitations were sent in August–September 2022. An additional letter was sent to the participants to communicate their identification code in the Vaud screening program because this information is mandatory to activate the participants' personal file in the screening program database. In addition to the mailed questionnaire T2, the participants who had not responded were contacted by the phone, which allowed us to collect 27 additional responses.

## RESULTS

### Participant characteristics

Of 6,200 mailed invitations, 5,396 went to a valid address, 1,059 individuals responded to the invitation letter (19.6% of valid addresses), and 946 signed the main consent form (17.5%). Among them, 318 were excluded because they did not meet inclusion criteria (Table 1), 87 because they responded after the end of the recruitment, 19 because they did not complete questionnaire 1, and 7 eligible participants were forgotten to invite (Figure 1 and Table 1). The remaining 515 participants were randomized to the intervention (n = 258) or the control group (n = 257). The mean age was 52.2 years (SD = 2.2), and the mean 15-year CRC risk was 1.4% (SD = 0.5). Stratification by the risk level showed that 98.1% (n = 505) of the participants were at low risk, 1.9% (n = 10) were at moderate risk, and 0 at high risk. Sociodemographic characteristics, family history of polyps or CRC, and the baseline intention to be screened were similar between intervention and control groups (Table 2). Postrandomization, 5 withdrawals were registered in the intervention group and 3 in the control group. Seven participants withdrew without giving any reason and 1 because of insufficient knowledge of French. An additional 2 participants were excluded postrandomization at T1 (1 from the intervention group and 1 from the control group) because they had done screening before receiving our intervention. Finally, 42 participants (16 in the intervention group and 26 in the control group) did not complete the follow-up questionnaire after reminders (Figure 1). Participants without data for the primary outcome were assumed to not have completed screening.

**Table 1.**
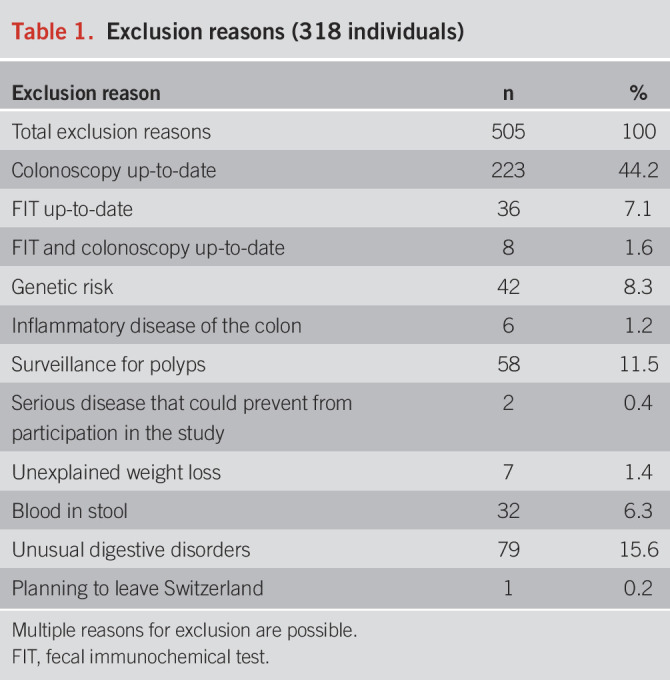
Exclusion reasons (318 individuals)

**Table 2.**
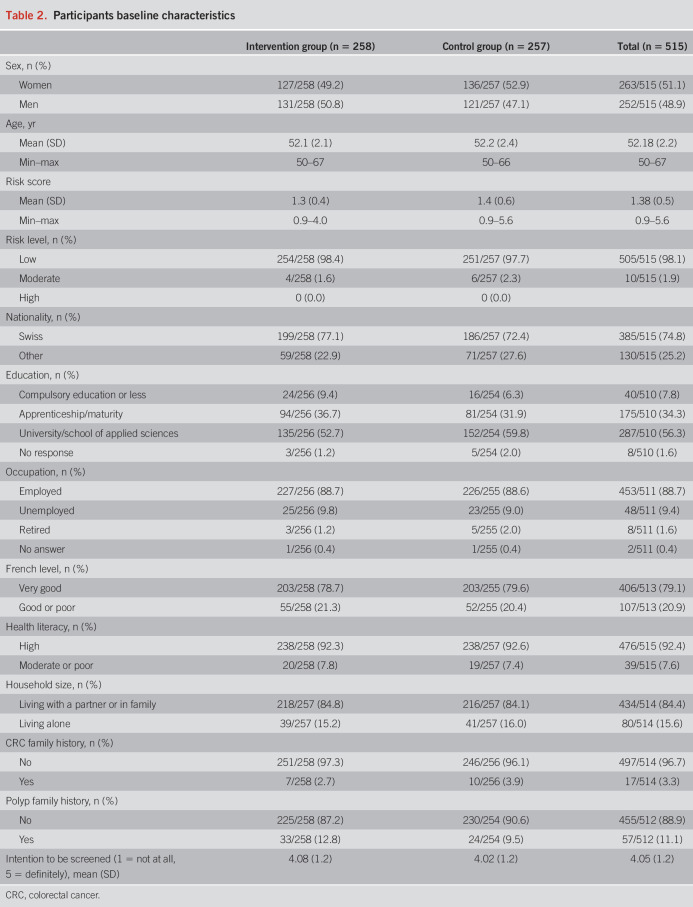
Participants baseline characteristics

### Primary outcome

At 6 months, 37% of participants who received the intervention underwent risk-appropriate screening compared with 23% in the control group (14% absolute increase, 95% confidence interval 6%–22%). Results are presented in Table 3. Exploratory subgroup analyses by sex, education, French level, household size, and family history of CRC and polyps did not reveal statistically significant heterogeneity in the effect of the intervention (see Supplementary Table 2, Supplementary Digital Content 2, http://links.lww.com/AJG/D530). There was a trend toward a greater effect of the intervention on women than men.

**Table 3.**
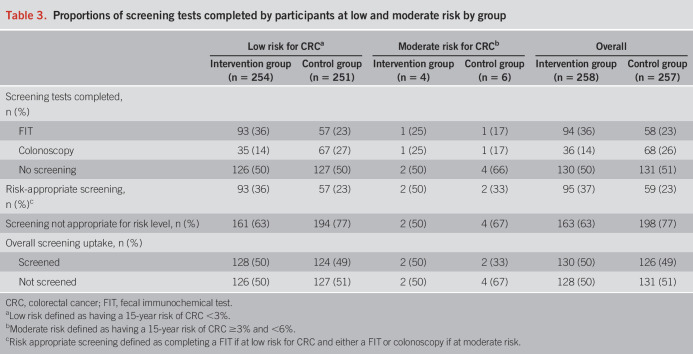
Proportions of screening tests completed by participants at low and moderate risk by group

### Secondary outcomes

Overall screening uptake was 50% in the intervention group and 49% in the control group (1% absolute difference, 95% confidence interval −0.07 to 0.1). Anxiety related to the intervention was low (intervention group: mean = 1.5 [SD = 0.5], control group: mean = 1.6 [SD = 0.5]), with no statistically significant difference between the 2 groups (*P* = 0.15). As the mean anxiety was low, no subgroup analysis was performed.

Our exploratory analyses by sex, education, French level, household size, and family history of CRC and polyps did not reveal significant differences in overall screening uptake between groups (see Supplementary Table 3, Supplementary Digital Content 2, http://links.lww.com/AJG/D530).

## DISCUSSION

This trial aimed at studying the effect of communicating personalized CRC risk and risk-appropriate screening recommendations on screening behavior. Participants who received the intervention were 14% more likely to undergo risk-appropriate screening compared with control group participants, who received materials presenting colonoscopy and FIT as equal options. Overall screening uptake was not affected by the intervention. These results support the use of risk-based screening recommendations to better allocate colonoscopy resources, particularly in areas offering direct screening colonoscopy, such as the United States and Germany (28).

Our main results are in line with Emery et al (12) in which risk appropriate screening increased by 21% after the intervention delivered in person by health professionals. In our study, the intervention consisted of a mailed brochure. We believe that for an intervention which did not require medical staff involvement, an increase of 14% in risk-appropriate screening is a promising result.

We reported a greater impact on appropriate screening than Skinner et al (13), who communicated CRC risk followed by multiple screening options. This suggests that recommending 1 screening test that corresponds to the individual's CRC risk is a promising strategy. It seems that short and tailored messages allow to avoid overwhelming participants with information and facilitate decision-making.

In our study, overall screening uptake did not differ between groups which is in line with earlier findings (7,8,13). It seems that communicating risk and screening recommendations are insufficient to enhance overall screening uptake, a result also seen in other cancer types (10). Other interventions such as mailed FIT and patient navigation are more effective for increasing screening rates (29).

Although the majority of our participants evaluated their intention to be screened as high, only 50% of them completed a screening test. Such intention-behavior gap is common and may be explained by a low priority of screening and overload in everyday life, or a need to discuss screening with a health professional. Previous studies also showed high level of nonparticipation among people with high intention for screening (30,31).

Results of our trial showed that communicating individual risk and screening recommendations were acceptable to participants and did not increase anxiety. In the context of organized screening programs, risk communication could be simplified by incorporating a specific module into a program's website allowing an automatic calculation of CRC risk and delivery of recommendations. Further studies should test the entire personalized screening pathway, from the collection of risk information to examination of the impact of risk-based screening on participation rates, the performance of the modified risk calculator, and the ability to detect advanced neoplasia. Such a trial, involving multiple screening programs, is planned for 2025 (32).

This is one of the first studies examining the impact of risk-based screening recommendations on risk-appropriate screening. By nesting the study in an organized program, we were able to recruit a representative portion of the target population and to test an intervention suitable for widespread adoption. Quality of the collected data was ensured by the institutional trial monitoring team, the study steering committee, and a citizen advisory group.

Our study did have limitations. Only individuals who had not yet been invited to the Program were recruited. This resulted in the inclusion of people with a mean age of 52 years old. Our previous modelling study showed that the QCancer risk of only 1.6% and 0.3% of Swiss males and females aged 50–54, respectively, exceeds 3% (21). Moreover, participants at high risk might already be up to date with screening or followed for colon conditions, and therefore ineligible to participate. This prevented us from testing the impact of our intervention on high-risk individuals.

Anxiety related to the intervention was low, which corroborated results of previous studies (9,10). Nevertheless, this result needs to be interpreted with caution, as majority of our participants were at low risk, and none at high risk.

We considered a colonoscopy appointment as a complete screening test, as in Switzerland missed appointments are rare (23). However, such assumption would not be appropriate in areas with high rates of missed colonoscopy appointments.

Our study materials were available only in French. Although the intervention and control brochures were written in plain language and approved by the citizen advisory group, people with limited French-language proficiency could have had difficulties participating.

Finally, our recruitment procedure required signing a consent form and completing questionnaires before receiving the study materials. This may have created a selection bias with inclusion of more motivated individuals. This would limit our ability to extrapolate our results to routine screening. However, our 19% response rate was similar to the 24% rate of participation after mailed invitation to the Vaud screening program among persons aged 50–54 years (16).

This trial demonstrated that an intervention brochure that communicates CRC risk and appropriate screening recommendations can increase risk-appropriate screening uptake among people at low risk without impacting the overall uptake rate. Future research should evaluate the impact of this approach on high-risk individuals and the impact of personalized screening on the detection of advanced neoplasia. The current results will be valuable for screening programs in Switzerland and other settings seeking to optimize the use of colonoscopy resources and decrease the burden of screening on participants.

## CONFLICT OF INTEREST

**Guarantor of the article:** Kevin Selby, MAS, MD.

**Specific author contributions:** Original concept of the study: K.S., J.-L.B., R.A., F.F., I.L.V., M.-A.D., and D.C. Funding acquisition: K.S. and J.-L.B. Drafting of the manuscript: K.S., E.P., and M.-A.D. Statistical analyses and randomization plan: A.C. All authors have reviewed and approved the manuscript before submission.

**Financial support:** This study was financed by a grant from the Swiss Cancer Research Foundation (KLS 5111-08-2020). Kevin Selby's salary was in part paid by the Fondation Leenaards.

**Potential competing interests:** M.-A.D. has contributed to the development of Option Grid patient decision aids. EBSCO Information Services sells subscription access to Option Grid patient decision aids. She receives consulting income from EBSCO Health and royalties. No other competing interests declared from her or the other investigators.

**Trial registration:** ClinicalTrials.gov (NCT05357508).

**Data sharing:** Deidentified individual participant data are available from the corresponding author on reasonable request.

**Study Highlights**

**WHAT IS KNOWN**

✓ Colorectal cancer mortality can be prevented or reduced by screening using colonoscopy or fecal immunochemical test (FIT).
✓ Personalized screening could optimize the use of colonoscopy resources, which is crucial to reduce screening burden for patients and society.
✓ The impact of personalized screening on screening behaviors is not clearly understood.

**WHAT IS NEW HERE**

✓ After reading our intervention brochure, participants were 14% more likely to choose the screening test appropriate to their risk level. This result did not affect overall screening uptake.
✓ Risk-based screening recommendations for FIT or colonoscopy could be a means of better allocating colonoscopy resources in countries relying heavily on colonoscopy for screening, thus decreasing the burden of colorectal cancer screening for low-risk participants.

## Supplementary Material
