# Supplementary figures and images for: Colorectal Cancer Screening Based on Predicted Risk: A Randomized Controlled Trial
Source: Am J Gastroenterol. 2025 Jan 7;120(10):2432–9. doi: 10.14309/ajg.0000000000003311 (PMC12487660; doi:10.14309/ajg.0000000000003311)

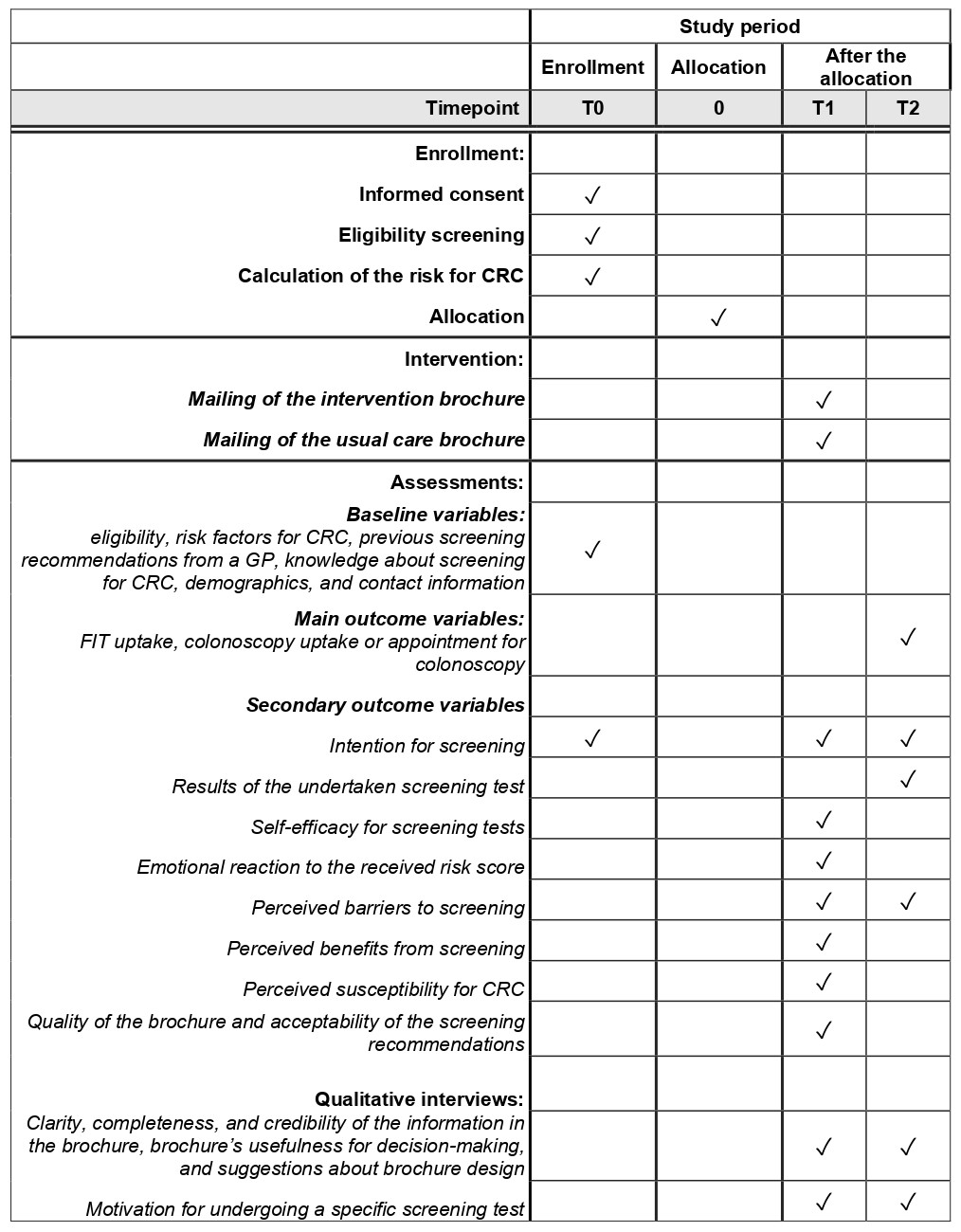

Supplement: Supplementary file 1 [file acg-120-2432-s001.jpg]
