# Supplementary material for: Colorectal Cancer Screening Based on Predicted Risk: A Randomized Controlled Trial
Source: Am J Gastroenterol. 2025 Jan 7;120(10):2432–9. doi: 10.14309/ajg.0000000000003311 (PMC12487660; doi:10.14309/ajg.0000000000003311)
Supplement: Supplementary file 2 [file acg-120-2432-s002.docx]

# Supplementary tables

Supplementary table 1. Types of information presented in the intervention and control brochures.

Supplementary table 2. Difference in risk-appropriate screening proportions by socioeconomic and CRC risk factors

Supplementary table 3. Difference in overall screening uptake by socioeconomic and CRC risk factors

Supplementary table 1. Types of information presented in the intervention and control brochures.

| Types of information | Intervention brochure | | | Control brochure |
| --- | --- | --- | --- | --- |
|  | High risk | Low risk | Moderate risk |  |
| Information about CRC | Yes | Yes | Yes | Yes |
| Information about screening benefits | Yes | Yes | Yes | Yes |
| Explanation concerning risk levels and how they can be calculated | Yes | Yes | Yes | No |
| Personalized risk score | Yes | Yes | Yes | No |
| Screening recommendations | Personalized: colonoscopy recommended; FIT the alternative | Personalized: FIT recommended; colonoscopy the alternative | Personalized: FIT and colonoscopy are equal options | General: FIT and colonoscopy are equal options |
| Explanations why recommended test(s) is(are) risk-appropriate | Yes | Yes | Yes | No |
| Short instructions about how uptake FIT / how to prepare bowel for colonoscopy | Yes, instructions for colonoscopy only | Yes, instructions for FIT only | Yes, instructions for both tests | Yes, instructions for both tests |
| Warning that risk increases with age and encouragements to maintain a healthy lifestyle | Yes | Yes | Yes | Non |
| Suggestion to consult a doctor if CRC symptoms occur | Yes | Yes | Yes | Yes |
| Information about cost coverage of screening tests | Yes | Yes | Yes | Yes |

Supplementary table 2. Difference in risk-appropriate screening proportions by socioeconomic and CRC risk factors

|  |  | Intervention group | | Control group | | Mean difference | | p-value for interaction |
| --- | --- | --- | --- | --- | --- | --- | --- | --- |
|  | n | Proportion | 95% CI | Proportion | 95% CI | Difference | 95% CI |  |
| Sex |  |  |  |  |  |  |  |  |
| Women | 263 | 0.43 | 0.34, 0.51 | 0.22 | 0.15, 0.29 | 0.2 | 0.09, 0.32 | 0.13 |
| Men | 252 | 0.31 | 0.23, 0.39 | 0.24 | 0.16, 0.32 | 0.07 | -0.04, 0.18 |  |
| Education |  |  |  |  |  |  |  |  |
| Compulsory school or less | 40 | 0.33 | 0.14, 0.52 | 0.31 | 0.09, 0.54 | 0.02 | -0.27, 0.32 | 0.43 |
| Apprenticeship/Maturity/High school/University | 462 | 0.37 | 0.31, 0.43 | 0.23 | 0.18, 0.29 | 0.14 | 0.06, 0.22 |  |
| French level |  |  |  |  |  |  |  |  |
| Very good | 406 | 0.35 | 0.28, 0.42 | 0.22 | 0.16, 0.27 | 0.13 | 0.05, 0.22 | 0.71 |
| Good or poor | 107 | 0.44 | 0.31, 0.57 | 0.25 | 0.13, 0.37 | 0.19 | 0.01, 0.36 |  |
| Household |  |  |  |  |  |  |  |  |
| Living with a partner or in family | 434 | 0.38 | 0.31, 0.44 | 0.21 | 0.15, 0.26 | 0.17 | 0.08, 0.25 | 0.06 |
| Living alone | 80 | 0.31 | 0.16, 0.45 | 0.34 | 0.2, 0.49 | -0.03 | -0.24, 0.17 |  |
| Family history of CRC and polyps |  |  |  |  |  |  |  |  |
| Yes | 72 | 0.31 | 0.16, 0.45 | 0.18 | 0.05, 0.31 | 0.13 | -0.07, 0.32 | 0.98 |
| No | 443 | 0.39 | 0.31, 0.44 | 0.24 | 0.18, 0.29 | 0.14 | 0.06, 0.23 |  |

Supplementary table 3. Difference in overall screening uptake by socioeconomic and CRC risk factors

|  |  | Intervention group | | Control group | | Mean difference | | p-value for interaction |
| --- | --- | --- | --- | --- | --- | --- | --- | --- |
|  | n | Proportion | 95% CI | Proportion | 95% CI | Difference | 95% CI |  |
| Sex |  |  |  |  |  |  |  |  |
| Women | 263 | 0.56 | 0.42, 0.65 | 0.49 | 0.4, 0.57 | 0.07 | -0.05, 0.19 | 0.18 |
| Men | 252 | 0.45 | 0.37, 0.54 | 0.5 | 0.41, 0.58 | -0.05 | -0.17, 0.08 |  |
| Education |  |  |  |  |  |  |  |  |
| Compulsory school or less | 40 | 0.33 | 0.14, 0.52 | 0.63 | 0.39, 0.86 | -0.29 | -0.59, 0.01 | 0.07 |
| Apprenticeship/Maturity/High school/University | 462 | 0.52 | 0.45, 0.58 | 0.5 | 0.43, 0.56 | 0.02 | -0.07, 0.11 |  |
| French level |  |  |  |  |  |  |  |  |
| Very good | 406 | 0.51 | 0.44, 0.58 | 0.46 | 0.39, 0.53 | 0.04 | -0.05, 0.14 | 0.23 |
| Good or poor | 107 | 0.49 | 0.36, 0.62 | 0.58 | 0.44, 0.71 | -0.08 | -0.27, 0.1 |  |
| Household |  |  |  |  |  |  |  |  |
| Living with a partner or in family | 434 | 0.5 | 0.43, 0.56 | 0.47 | 0.4, 0.53 | 0.03 | -0.07, 0.12 | 0.41 |
| Living alone | 80 | 0.54 | 0.38, 0.69 | 0.61 | 0.46, 0.76 | -0.07 | -0.29, 0.14 |  |
| Family history of CRC and polyps |  |  |  |  |  |  |  |  |
| Yes | 72 | 0.59 | 0.44, 0.74 | 0.58 | 0.41, 0.74 | 0.01 | -0.22, 0.24 | 0.98 |
| No | 443 | 0.49 | 0.42, 0.55 | 0.48 | 0.41, 0.54 | 0.01 | -0.08, 0.1 |  |
